# Supplementary material for: MetaQTL: a package of new computational methods for the meta-analysis of QTL mapping experiments
Source: BMC Bioinformatics. 2007 Feb 8;8:49. doi: 10.1186/1471-2105-8-49 (PMC1808479; doi:10.1186/1471-2105-8-49)
Supplement: Additional File 7 — MetaQTL Package : jar file and tutorial. This Zip archive contains both the MetaQTL JAR file and the files of the tutorial. [file 1471-2105-8-49-S7.zip › org.inra.metaqtl/doc/org/metaqtl/main/class-use/CmdLineParser.IllegalOptionValueException.html]

Uses of Class org.metaqtl.main.CmdLineParser.IllegalOptionValueException


|  |  |  |
| --- | --- | --- |
| **Overview** \| **Package** \| **Class** \| **Use** \| **Tree** \| **Deprecated** \| **Index** \| **Help** | |  |
| PREV   NEXT | **FRAMES**    **NO FRAMES**     **All Classes** |  |


---


## **Uses of Class org.metaqtl.main.CmdLineParser.IllegalOptionValueException**

| Packages that use CmdLineParser.IllegalOptionValueException | |
| --- | --- |
| **org.metaqtl.main** |  |

Uses of CmdLineParser.IllegalOptionValueException in org.metaqtl.main

| Methods in org.metaqtl.main that throw CmdLineParser.IllegalOptionValueException | |
| --- | --- |
| `java.lang.Object` | `CmdLineParser.Option.getValue(java.lang.String arg, java.util.Locale locale)` |
| `void` | `CmdLineParser.parse(java.lang.String[] argv)`             Extract the options and non-option arguments from the given list of command-line arguments. |
| `void` | `CmdLineParser.parse(java.lang.String[] argv, java.util.Locale locale)`             Extract the options and non-option arguments from the given list of command-line arguments. |
| `protected  java.lang.Object` | `CmdLineParser.Option.parseValue(java.lang.String arg, java.util.Locale locale)`             Override to extract and convert an option value passed on the command-line |
| `protected  java.lang.Object` | `CmdLineParser.Option.IntegerOption.parseValue(java.lang.String arg, java.util.Locale locale)` |
| `protected  java.lang.Object` | `CmdLineParser.Option.LongOption.parseValue(java.lang.String arg, java.util.Locale locale)` |
| `protected  java.lang.Object` | `CmdLineParser.Option.DoubleOption.parseValue(java.lang.String arg, java.util.Locale locale)` |

---


|  |  |  |
| --- | --- | --- |
| **Overview** \| **Package** \| **Class** \| **Use** \| **Tree** \| **Deprecated** \| **Index** \| **Help** | |  |
| PREV   NEXT | **FRAMES**    **NO FRAMES**     **All Classes** |  |


---
